# Serum renin levels refine acute kidney injury prediction in critically ill children

**DOI:** 10.1007/s00467-025-07061-0

**Published:** 2025-11-22

**Authors:** Naomi Pode-Shakked, Giovanni Ceschia, James E. Rose, Kelli A. Krallman, Stuart L. Goldstein, Natalja L. Stanski

**Affiliations:** 1 Cincinnati Children’s Hospital Medical Center, 3333 Burnet Ave, Cincinnati, OH 45208 USA; 2 Pediatric Nephrology Unit, Dana Dwek Children’s Hospital, Tel Aviv Medical Center, Tel Aviv, Israel; 3 Gray Faculty of Medical & Health Sciences, Tel-Aviv University, Tel Aviv, Israel; 4 Pediatric Nephrology Unit, Department of Women’s and Children’s Health, University-Hospital of Padova, Via Giustiniani 3, 35128 Padua, Italy; 5 Department of Pediatrics, University of Cincinnati College of Medicine, 3230 Eden Ave, Cincinnati, OH 45267 USA

**Keywords:** Acute kidney injury, Renin, Biomarkers, Risk stratification, Precision medicine

## Abstract

**Background:**

Studies demonstrate that elevated renin is associated with adverse outcomes in critical illness. We aimed to evaluate whether serum renin enhances acute kidney injury (AKI) risk stratification in critically ill children.

**Methods:**

A prospective, observational pilot study of PICU patients from the TAKING FOCUS 2 (TF2) study for whom direct renin levels were measured within 48 h of PICU admission. TF2 employed the Renal Angina Index (RAI) (RAI +  ≥ 8) and urine neutrophil gelatinase-associated lipocalin (uNGAL; uNGAL +  ≥ 150 ng/mL) to aid in the risk prediction of severe AKI (sAKI; ≥ KDIGO stage 2) at PICU day 2–4. We examined renin levels across TF2 algorithm branchpoints, assessed the additive predictive performance of renin ≥ 100 pg/mL for sAKI, and assessed associations between elevated renin and outcomes.

**Results:**

Among 107 patients (53% male, median age 8 [2–15] years), 30 (28%) were RAI–, 77 (72%) were RAI+ , and 43 (40%) had sAKI. Median renin concentration was 61.3 [16.5–143.8] pg/mL, increasing progressively across sAKI risk strata: RAI+  > RAI– (70.4 [24.7–182.1] vs. 33.3 [11.2–93.9] pg/mL,* p* = 0.006) and RAI+ /uNGAL +  > RAI+ /uNGAL– (103.7 [47–507] vs. 42.1 [15.9–125] pg/mL,* p* = 0.01). Patients with sAKI had higher renin (102 [35.2–374] vs. 41.6 [11.4–111] pg/mL, *p* = 0.002), including after adjustment for covariates (*p* = 0.001). Renin ≥ 100 pg/mL was independently associated with mortality (aOR 4.0, 95% CI 1.06–14.9, *p* = 0.041). Adding renin ≥ 100 pg/mL to RAI+ /uNGAL+ improved specificity (93% from 84%) and PPV (81% from 77%) of day 2–4 sAKI prediction.

**Conclusions:**

Serum renin levels increase progressively across sAKI risk strata and appear to enhance sAKI prediction.

**Graphical abstract:**

**Figure:**
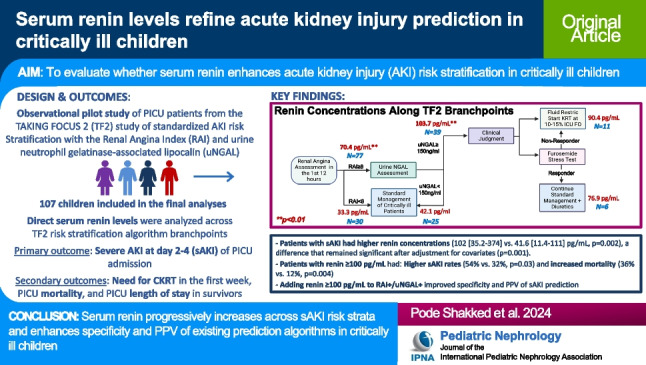
A higher resolution version of the Graphical abstract is available as Supplementary information

**Supplementary Information:**

The online version contains supplementary material available at 10.1007/s00467-025-07061-0.

## Introduction

Acute kidney injury (AKI) is a frequent complication in critically ill children and contributes to increased morbidity and mortality [1–4]. Given the limited treatment options, early and accurate risk stratification for severe AKI (sAKI) (Kidney Disease Improving Global Outcomes [5] stage 2 or 3) is important, as it enables the implementation of targeted renal-protective interventions that may improve outcomes [6–8]. The Renal Angina Index (RAI) is a clinical tool that has been validated to aid in the risk assessment for new or persistent sAKI on pediatric intensive care unit (PICU) day 3 in critically ill children with high sensitivity and negative predictive value [6, 7, 9–12]. The addition of directed measurement of tubular injury biomarkers like urinary neutrophil gelatinase-associated lipocalin (uNGAL) has also been shown to refine this predictive ability [8].

The Trial in AKI using NGAL and Fluid Overload to optimize CRRT Use (TAKING FOCUS 2; TF2) was a single-center study that employed sequential risk stratification with the RAI and uNGAL to inform non-mandatory clinical decision support (CDS) around fluid management, including loop diuretic challenges and continuous kidney replacement therapy (CKRT) use (Figure S1) [13]. The TF2 paradigm employs sequential risk assessment, with the RAI identifying patients at risk for sAKI who undergo reflexive uNGAL measurement for further risk stratification. While this approach demonstrates excellent negative predictive values (NPVs) for sAKI (97–99%), the positive predictive values (PPVs) remain suboptimal (36–62%) [8, 11, 14, 15], representing an opportunity for algorithm refinement.

Recent studies in adults have highlighted the role of the renin–angiotensin–aldosterone system (RAAS) derangement, and specifically elevated renin levels, in critical illness and AKI [16, 17]. For example, Bellomo and colleagues found that renin levels > 173 pg/mL were associated with an increased risk of AKI, the need for KRT, and mortality in adult patients with vasodilatory shock [18, 19]. We similarly demonstrated that elevated serum renin and prorenin concentrations are associated with persistent sAKI and mortality in children with septic shock [20]. Mechanistically, decreased angiotensin-converting enzyme activity has been associated with adverse kidney outcomes in this population [21, 22], providing insight into the pathophysiological basis for elevated renin as a biomarker of RAAS dysregulation. Taken together, these data suggest that renin has the potential to enhance the specificity and PPV of existing AKI prediction algorithms in critically ill children.

Thus, we conducted a single-center observational pilot study that leveraged the existing TF2 algorithm to examine the additive predictive value of renin concentrations for sAKI. We also compared renin levels across TF2 algorithm branch points (Figure S1) and determined the association between elevated renin concentrations and clinical outcomes. We hypothesized that elevated renin levels would be associated with worse outcomes and would enhance TF2 algorithm prediction of day 2–4 sAKI.

## Methods

### Study design and population

This prospective observational pilot study was conducted in the PICU at Cincinnati Children’s Hospital Medical Center (CCHMC) from 3/2019 to 3/2023. The TF2 study (NCT03541785) was approved by the CCHMC Institutional Review Board with a waiver of informed consent (IRB no: 2018-0724, approved 02/2018), and complete methodological details have been previously published [10, 13, 23]. For this pilot study, we aimed to enroll at least 25 patients in each main arm of the TF2 pathway (RAI–, RAI+/uNGAL+, RAI+/uNGAL–, Figure S1) to ensure adequate representation across risk strata. Renin was measured from residual blood samples obtained for a standard of clinical care complete blood count test performed Monday through Friday between 8 AM and 5 PM in PICU-admitted patients. Patients from the original TF2 study were included in this pilot study if they had a sample available for serum renin measurement within 48 h of PICU admission (*n* = 107). Procedures were followed in accordance with the ethical standards of the responsible committee on human experimentation and with the 1964 Declaration of Helsinki and its later amendments. Study methods and results are reported in line with the Strengthening the Reporting of Observation studies in Epidemiology (STROBE) guidelines.

### Data collection and definitions

Demographic, clinical, and laboratory data were collected prospectively until day 7 or PICU discharge, whichever came first. Outcome data were tracked until day 28. AKI was defined according to the KDIGO criteria, with sAKI defined as stage 2 or higher by serum creatinine criteria [5]. Severity of illness was quantified on admission using the Pediatric Risk of Mortality Score III (PRISM III) [24] and daily using the vasoactive inotropic score (VIS) [25]. Sepsis was defined as two or more systemic inflammatory response syndrome (SIRS) criteria plus suspicion for infection [26]. Cumulative fluid accumulation was calculated daily as a percentage of fluid accumulation relative to admission weight, as has been previously done [23].

### TF2 pathway and biomarker assessment

A general schematic of the TF2 pathway is shown in Figure S1. The RAI was calculated 12 h after PICU admission using typical methods (Figure S2) [9]. An RAI ≥ 8 was considered positive for renal angina (RAI+), indicating increased risk for day 2–4 sAKI [6, 7, 9, 23, 27–29]. RAI+ patients were further risk stratified using reflexively measured uNGAL concentrations. uNGAL was quantified using the NGAL Test™ (Bioporto Diagnostics, Denmark) with values ≥ 150 ng/mL considered high risk for sAKI (uNGAL+). uNGAL values were missing for 13 of the RAI+ patients. Finally, as part of TF2, a subset of high-risk patients (RAI+/uNGAL+) underwent a furosemide stress test (FST) [30]. After ensuring adequate intravascular volume, patients received intravenous furosemide (1 mg/kg for furosemide-naïve patients or 1.5 mg/kg for patients on prior furosemide therapy), and urine output was measured hourly for 4 h following administration. Patients were classified as FST responders if they produced ≥ 3 mL/kg/h of urine within the 4-h period; otherwise, they were classified as non-responders [31].

Serum direct renin was measured via the DRG Renin ELISA Kit (DRG International Inc., Springfield, NJ, USA) using opportunistic samples collected within 48 h of the RAI result. A direct renin threshold of 100 pg/mL was selected a priori for primary analyses. This value is roughly twice the upper limit of the normal range for healthy children and adolescents, where median values are typically 25–45 pg/mL and the 98^th^ percentile rarely exceeds 50–60 pg/mL [32, 33]. This threshold also aligns well with thresholds identified in adult ICU studies demonstrating that renin values above 87–130 pg/mL predict shock, AKI, and mortality in diverse ICU populations [20, 34–36].

### Outcomes

The primary aim of this study was to describe differences in serum renin concentrations across the TF2 sAKI risk stratification algorithm. Thus, the primary outcome examined was day 2–4 sAKI. Secondary outcomes included the need for CKRT in the first week, PICU mortality, and PICU length of stay (LOS) in survivors.

### Statistical analysis

Continuous variables were reported as median with interquartile range (IQR) and compared using the Mann–Whitney *U* test due to non-normal distribution. Categorical variables were reported as counts and percentages and compared using the chi-square test with Yates’ correction applied when any cell frequency was < 5.

Comparisons were first made between patients who experienced day 2–4 sAKI and those who did not, and between those with renin concentrations ≥ 100 pg/mL and < 100 pg/mL. Next, renin concentrations were compared at each branch point of the TF2 algorithm: RAI+ vs. RAI–; RAI+/uNGAL+ vs. RAI+/uNGAL–; FST responder vs. FST non-responder. Sensitivity, specificity, PPV, NPV, positive likelihood ratio (+LR), and negative likelihood ratio (–LR) were calculated to assess the predictive abilities of the RAI alone, RAI/uNGAL, and RAI/uNGAL/Renin for day 2–4 sAKI and CKRT use in the first week. Renin concentrations were then assessed across a variety of biologically relevant covariates with the potential to impact the RAAS pathway: age, need for vasoactive support, and sepsis. Each covariate was first dichotomized, and renin levels were compared between the resulting groups. Covariates that demonstrated significant associations with renin levels were incorporated into a multivariable linear regression model using log-transformed renin as the dependent variable to control for confounding effects. Residuals from this adjusted model, representing renin levels corrected for covariates, were then compared between patients with and without day 2–4 sAKI. Finally, multivariable logistic regression analysis was performed to assess the independent association between renin ≥ 100 pg/mL and mortality, adjusting for severity of illness by PRISM III score and need for vasoactive medications. Statistical significance was set at *p* < 0.05. All analyses were performed using R software (R Foundation for Statistical Computing, Vienna, Austria).

## Results

### Study population and baseline characteristics

A total of 107 patients were included in our analysis based on the availability of residual blood samples for renin measurement within 48 h of PICU admission. Thirty patients (28%) were RAI– and 77 (72%) were RAI+. Among RAI+ patients, 39 were RAI+/uNGAL+ (51%) and 25 were RAI+/uNGAL– (32%), and uNGAL measurements were missing for 13 (17%). Forty-three patients (40%) developed the primary outcome of day 2–4 sAKI. Cohort demographics, characteristics and outcomes by the presence or absence of day 2–4 sAKI are outlined in Table 1. There was an even distribution of patients within each biological sex (53% male, 47% female), and the median age of the cohort was 8 [2–15] years. Just over half (53%) had sepsis at admission, the median PRISM III score was 9 [5–15], the median VIS at admission was 0 (0–20), and 43 (40%) were receiving invasive mechanical ventilation (IMV). The median direct renin concentration for the cohort was 61.3 [16.5–143.8] pg/mL. Overall, 11 patients (10%) received CKRT in the first week of PICU admission and 18 (17%) died, with a median PICU LOS in survivors of 5 [2–12] days.

**Table 1:** Demographics, admission characteristics, and outcomes by the presence or absence of day 2–4 severe acute kidney injury

	All (*n* = 107)	No sAKI (*n* = 64)	sAKI (*n* = 43)	*p*-value
Biological sex				
Female, *n* (%) Male, *n* (%)	50 (47%) 57 (53%)	27 (42%) 37 (58%)	23 (53%) 20 (47%)	0.56
Age, years	8 (2–15)	9 (2–14)	8 (2–16)	0.46
PRISM III	9 (5–15)	8 (4–11)	10 (7–19)	0.02
Admission diagnosis				0.39
Shock	48 (45%)	25 (39%)	23 (53%)	
Medical cardiac	16 (15%)	11 (17%)	5 (12%)	
Respiratory failure	31 (29%)	18 (28%)	13 (30%)	
Post-surgical/minor trauma	34 (32%)	23 (36%)	11 (26%)	
CNS dysfunction	8 (7%)	7 (11%)	1 (2%)	
Pain/sedation management	2 (2%)	1 (2%)	1 (2%)	
Admission VIS	0 (0–10)	0 (0–8)	6 (0–20)	0.23
Sepsis at admission, yes (%)	57 (53%)	32 (50%)	25 (58%)	0.41
Admission IMV, yes (%)	43 (40%)	35 (55%)	22 (51%)	0.72
RAI score RAI+, *n* (%)	10 (6–22) 77 (72%)	8 (2–14) 35 (55%)	20 (8–32) 42 (98%)	** < **0.001 0.02
uNGAL (ng/mL)	204 (50–1577)	87 (50–217)	771 (197–2573)	** < **0.001
Direct renin (pg/mL)	61.3 (16.5–143.8)	41.6 (11.4–110.7)	102 (35.2–374.2)	0.002
CKRT day 1–7, yes (%)	11 (10%)	0 (0%)	11 (26%)	** <** 0.001
PICU mortality, yes (%)	18 (17%)	6 (10%)	12 (28%)	0.01
PICU LOS in survivors, days	5 (2–12)	5 (2–9)	6 (4–16)	0.02

Continuous data are presented as median (IQR)

*sAKI*, day 2–4 severe AKI; *PRISM III*, Pediatric Risk of Mortality III; *VIS*, vasoactive-inotropic score; *IMV*, invasive mechanical ventilation; *RAI*, renal angina index; *uNGAL*, urinary neutrophil gelatinase-associated lipocalin; *CKRT*, continuous kidney replacement therapy; *LOS*, length of stay

Patients who developed Day 2–4 sAKI had a higher severity of illness on admission by PRISM III score (10 [7–19] vs. 8 [4–11], *p* = 0.02), higher RAI scores (20 [8–32] vs. 8 [2–14], *p* < 0.001), and higher uNGAL (771 [197–2573] vs. 87 [50–217] ng/ml, *p* < 0.001) and direct renin (102 [35.2–374.2] vs. 41.6 [11.4–110.7] pg/mL, *p* = 0.002) concentrations. Patients who developed day 2–4 sAKI also received CKRT more commonly (26% vs. 0, *p* = 0.001) and experienced higher PICU mortality rates (28% vs. 10%, *p* = 0.01), while those who survived had a longer PICU LOS (6 [4–16] vs. 5 [2–9] days, *p* = 0.02).

#### Renin levels across TF2 algorithm

**Fig. 1:**
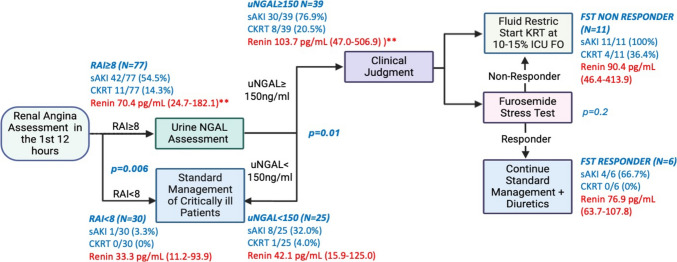
Incorporation of direct renin into the TAKING FOCUS 2 algorithm. Serum renin median (IQR) and rates of day 2–4 severe acute kidney injury (sAKI) and continuous kidney replacement therapy (CKRT) across each algorithm branchpoint. ** represents a significant difference (*p* < 0.05) in renin values between the two groups

Renin concentrations across the TF2 branch points are outlined in Fig. 1. The median time from PICU admission to renin measurement was 21.4 h (IQR 12.9–30.2). In general, renin concentrations were higher with increasing risk for day 2–4 sAKI, with RAI+ patients demonstrating higher median renin concentrations (70.4 [24.7–182.1] vs. 33.3 [11.2–93.9] pg/mL, *p* = 0.006). When RAI+ patients were further risk stratified by uNGAL, RAI+/uNGAL+ similarly demonstrated higher median renin concentrations compared to RAI+/uNGAL– (103.7 [47.0–506.9] vs. 42.1 [15.9–125.0] pg/mL, *p* = 0.01). These increases in renin concentration also paralleled the increased occurrence of day 2–4 sAKI, which occurred in 1 (3.3%) RAI–, 8 (32%) RAI+/uNGAL–, and 30 (77%) RAI+/uNGAL+ patients, respectively (Fig. 1). There was no difference noted in median renin levels in FST responders compared to non-responders (70.6 [63.7–107.8] pg/mL vs. 90.4 [46.4–413.9] pg/mL, *p* = 0.2).

#### Clinical outcome associations between elevated renin levels and impact on TF2 algorithm performance

A comparison of demographics, clinical characteristics and outcomes for patients dichotomized above and below a renin concentration of 100 pg/mL is outlined in Table 2. Patients with direct renin ≥ 100 pg/mL (*n* = 41, 38%) had higher severity of illness by PRISM III (11 [7–19] vs. 7 [4–12], *p* = 0.004) and admission VIS (5 [0–19] vs. 0 [0–5], *p* = 0.006), more commonly had sepsis (66% vs. 45%, *p* = 0.04), had greater admission risk for day 2–4 sAKI with higher RAI scores (12 vs. 8,* p* = 0.005) and uNGAL concentrations (469 [136–2284] vs. 128 [50–1204] ng/mL, *p* = 0.04), and had a greater peak fluid accumulation in the first week (9% vs. 7%,* p* = 0.02). These patients also had higher day 2–4 sAKI rates (54% vs. 32%,* p* = 0.03) and increased PICU mortality (30% vs. 9%,* p* = 0.005). The association between mortality and renin ≥ 100 pg/mL was maintained on multivariable logistic regression analysis adjusting for vasoactive use and PRISM III score (aOR 4.0, 95% CI 1.06–14.9, *p* = 0.041).

**Table 2:** Demographics, admission characteristics, and outcomes by direct renin concentration

	Renin < 100 pg/mL (***n*** = 66)	Renin ≥ 100 pg/mL (***n*** = 41)	***p***-value
Biological sex			
Female, *n* (%) Male, *n* (%)	32 (48%) 34 (52%)	18 (44%) 30 (56%)	0.24
Age, years	10 (2–16)	4 (1–14)	0.08
PRISM III	7 (4–12)	11 (7–19)	0.004
Admission diagnosis, *n* (%)			0.12
Shock	26 (39%)	22 (54%)	
Medical cardiac	6 (9%)	10 (24%)	
Respiratory failure	19 (29%)	12 (29%)	
Post-surgical/minor trauma	25 (38%)	9 (22%)	
CNS dysfunction	6 (9%)	2 (5%)	
Pain/sedation management	2 (3%)	0 (0%)	
Admission VIS	0 (0–5)	5 (0–19)	0.006
Sepsis at admission, yes (%)	30 (45%)	27 (66%)	0.04
Admission IMV, yes (%)	28 (42%)	29 (71%)	0.004
RAI score RAI+, *n* (%)	8 (4–20) 43 (65%)	12 (8–24) 34 (83%)	0.005 0.047
uNGAL (ng/ml)	128 (50–1204)	469 (136–2284)	0.04
Day 1–7 peak %fluid accumulation	7 (3–13)	9 (5–17)	0.02
Day 2–4 sAKI, yes (%)	21 (32%)	22 (54%)	0.03
CKRT day 1–7, yes (%)	5 (8%)	6 (15%)	0.24
PICU mortality, yes (%)	6 (9%)	12 (30%)	0.005
PICU LOS in survivors, days	5 (2–10)	5 (3–18)	0.12

Continuous data are presented as median (IQR)

*sAKI*, day 2–4 severe AKI; *PRISM III*, Pediatric Risk of Mortality III; *VIS*, vasoactive-inotropic score; *IMV*, invasive mechanical ventilation; *RAI*, renal angina index; *uNGAL*, urinary neutrophil gelatinase-associated lipocalin; *CKRT*, continuous kidney replacement therapy; *LOS*, length of stay

The predictive performance of each component of the TF2 algorithm (RAI+ alone, RAI+/uNGAL+, RAI+/uNGAL+/Renin+) is outlined in Table 3. In general, across these risk strata, sensitivity and NPV begin high and progressively decrease with the addition of uNGAL and renin, while specificity and PPV start low and increase with each addition. Specifically, the addition of Renin+ to the RAI+/uNGAL+ paradigm increased the specificity of day 2–4 sAKI prediction from 84 to 93% and PPV from 77 to 81%, while reducing sensitivity from 77 to 41% and NPV from 84 to 69%. Similarly, adding Renin+ to RAI+/uNGAL+ increased the specificity of the prediction of CKRT use from 64 to 82%, while reducing sensitivity from 89 to 45%. Changes in PPV and NPV were less notable for this outcome given the infrequency of its occurrence.

**Table 3:** Sequential performance of progressive risk stratification for day 2–4 severe acute kidney injury and continuous kidney replacement therapy use across the TAKING FOCUS 2 algorithm and with addition of renin ≥ 100 pg/mL

	RAI+ (*n* = 77)	RAI+/uNGAL+ (*n* = 39)	RAI+/uNGAL+/Renin+ (*n* = 21**)**
Day 2–4 sAKI Sensitivity Specificity PPV NPV +LR –LR	42/77 (55%) 98% (87–100) 45% (33–58) 55% (49–60) 97% (80–100) 1.8 (1.4–2.2) 0.05 (0.01–0.36)	30/39 (77%) 77% (61–89) 84% (71–92) 77% (64–86) 84% (74–90) 4.7 (2.5–8.8) 0.28 (0.15–0.50)	17/21 (81%) 41% (26–57) 93% (84–98) 81% (61–92) 69% (63–74) 6.1 (2.2–16.8) 0.6 (0.5–0.8)
CKRT use Sensitivity Specificity PPV NPV +LR –LR	11/77 (14%) 100% (72–100) 31% (22–42) 14% (13–16) 100% (88–100) 1.5 (1.3–1.7) 0.0 (0.0–0.0)	8/39 (21%) 89% (52–100) 64% (52–74) 21% (15–27) 98% (89–100) 2.4 (1.7–3.5) 0.17 (0.0–1.1)	5/21 (24%) 45% (17–77) 82% (73–90) 24% (12–41) 93% (88–96) 2.6 (1.2–5.7) 0.7 (0.4–1.1)

*sAKI*, severe AKI; *PPV*, positive predictive value; *NPV*, negative predictive value; +*LR*, positive likelihood ratio; *–LR*, negative likelihood ratio; *RAI*, renal angina index; *uNGAL*, urinary neutrophil gelatinase-associated lipocalin; *CKRT*, continuous kidney replacement therapy

### Renin levels across biologically relevant covariates and impact on association with day 2–4 sAKI

Lastly, we assessed differences in serum renin concentrations across several relevant biological covariates that could potentially influence the RAAS (Table 4). In this cohort, there were no differences in renin concentrations between the two biological sexes. However, differences were seen across age strata, with younger children (< 8 years old or median age of cohort) having higher renin concentrations (88.1 [29.3–158.2] vs. 41.6 [11.0–112.7] pg/mL in children ≥ 8, *p* = 0.02). Higher renin concentrations were also noted in children with sepsis (82.3 [29.3–158.2] vs. 41.6 [9.7–122.6] pg/mL, *p* = 0.009) and in those requiring vasoactive support (109.3 [45.1–506.9] vs. 44.1 [10.7–108.9] pg/mL, *p* < 0.001). Based on these results, we performed multivariable regression analysis to assess the independent association between renin and the primary outcome of day 2–4 sAKI, after adjusting for these covariates (Table 4). The association between elevated renin levels and day 2–4 sAKI was maintained after individual adjustment for vasoactive support (*p* < 0.001), age < 8 years (*p* = 0.005), and sepsis (*p* = 0.006) and in a model including all three covariates (*p* = 0.001) (Table 4).

**Table 4:** Renin levels compared across biologically relevant covariates that may impact renin–angiotensin–aldosterone (RAAS) and adjustment for day 2–4 severe AKI associations

Variable	Group	N	Renin (pg/mL), median (IQR)	*p*-values
Day 2–4 sAKI	No	64	41.6 (11.4–110.7)	0.002
	Yes	43	102.0 (35.2–374.2)	
Sex	Male	57	63.0 (11.9–128.2)	0.39
	Female	50	59.6 (20.7–155.5)	
Age	< 8 years	53	88.1 (29.3–158.2)	0.02*
	≥ 8 years	54	41.6 (11.0–112.7)	
Sepsis	No	50	41.6 (9.7–122.6)	0.009*
	Yes	57	82.3 (29.3–158.2)	
VIS on the day of renin measurement	0	63	44.1 (10.7–108.9)	< 0.001*
	> 0	35	109.3 (45.1–506.9)	
Adjusted comparison of renin levels between those with and without day 2–4 sAKI				
Unadjusted				0.002
Adjusted for VIS > 0				< 0.001
Adjusted for age < 8				0.005
Adjusted for sepsis				0.006
Adjusted for all variables (VIS > 0, AGE < 8, sepsis)				0.001

*sAK*I, severe *AKI*; *VIS*, vasoactive-inotropic score

*Included covariates in multivariable model

## Discussion

In this prospective observational pilot study of critically ill children, we demonstrate that serum direct renin concentrations progressively increase across sAKI risk strata and enhance the specificity and PPV of sAKI prediction when combined with the existing TF2 framework. We also demonstrate that elevated renin levels within 48 h of PICU admission are associated with poor outcomes, including day 2–4 sAKI and PICU mortality. Importantly, the association with both outcomes persisted after adjustment for relevant covariates. These findings extend our previous observations in pediatric septic shock to the broader PICU population and provide preliminary support for the hypothesis that RAAS dysregulation serves as a clinically relevant biomarker for sAKI in critically ill children [17, 20, 34, 35].

The strong association between elevated renin and day 2–4 sAKI in our cohort aligns with emerging evidence in both adult and pediatric critical care populations [16, 18, 20–22]. Our findings parallel those of Bellomo, who demonstrated that renin levels > 173 pg/mL were associated with increased risk of AKI, the need for kidney replacement therapy, and mortality in adults with vasodilatory shock [18]. Similarly, Flannery reported that elevated renin was independently associated with major adverse kidney events in a multicenter cohort of critically ill adults [16]. In the pediatric population, our results build upon previous work showing that serum renin and prorenin concentrations predict severe persistent AKI and mortality in pediatric septic shock [20], as well as findings by Kuai demonstrating that early urinary renin excretion is associated with stage 3 AKI and mortality in the PICU [22]. The association between renin and sAKI in our study remained after adjustment for key confounders including age, sepsis, and vasoactive support, suggesting that renin elevation reflects specific pathophysiological processes related to kidney injury rather than merely serving as a marker of illness severity. This independent association supports the mechanistic basis for RAAS dysregulation in pediatric AKI, as previously demonstrated by our group’s findings that decreased angiotensin-converting enzyme activity is associated with adverse kidney outcomes in children with septic shock [21].

The clinical utility of renin as a predictive biomarker for sAKI lies in its ability to improve the specificity and PPV of existing prediction algorithms. While the TF2 algorithm demonstrates excellent NPVs (97–99%), its PPV remains suboptimal at 36–62% [8, 11, 14, 15], leading to potential overtreatment and resource utilization concerns. Our study demonstrates that incorporating renin ≥ 100 pg/mL into the RAI+/uNGAL+ cohort increases specificity to 93% and PPV to 81%, effectively reducing false positives while maintaining reasonable sensitivity. This improvement has additional clinical implications for resource allocation and intervention planning. In a healthcare environment where PICU beds, CKRT, and intensive monitoring resources are limited, the ability to more accurately identify patients at highest risk for sAKI could lead to more targeted and cost-effective care. Furthermore, patients identified by the enhanced algorithm (RAI+/uNGAL+/Renin+) may represent a population that would benefit from emerging targeted therapies, such as angiotensin II agonists, which have shown promise at mitigating AKI in adult populations with RAAS dysregulation [18, 19, 37]. Finally, the progressive increase in renin concentrations across TF2 algorithm branches (RAI+  > RAI–, RAI+/uNGAL+  > RAI+/uNGAL–) suggests that renin elevation parallels the severity of underlying kidney injury because of RAAS dysregulation and is an important area for future mechanistic studies. Ultimately, larger, multi-center studies are needed to validate these findings before the clinical utility of renin as a biomarker for sAKI can be elucidated.

Beyond its utility in AKI prediction, our study revealed that elevated renin levels (≥ 100 pg/mL) were associated with higher mortality rates, extending its prognostic value beyond kidney-specific outcomes. These results are consistent with adult studies demonstrating relationships between renin and mortality in critical illness [17, 34, 35]. Gleeson showed that renin serves as a marker of tissue perfusion and prognosis in critically ill adults, with elevated levels predicting worse outcomes [34]. Similarly, Lesnik demonstrated that renin is a marker of tissue perfusion, septic shock, and mortality in septic patients [35]. Our previous work in pediatric septic shock similarly demonstrated that elevated serum renin and prorenin concentrations predict mortality, with the trajectory of renin levels from day 1 to day 3 providing additional prognostic information about clinical deterioration [20]. These findings, along with those reported from the current study, suggest that RAAS dysregulation may represent a key pathophysiological pathway connecting organ dysfunction and clinical outcomes across age groups. This broader prognostic utility supports the potential for renin to serve not only as an AKI biomarker, but as an indicator of overall illness trajectory and a guide for escalation of care decisions in critically ill children.

Our study has several important strengths and limitations. Strengths include the prospective design, standardized AKI definitions using KDIGO criteria, integration of renin with established biomarkers within a validated CDS algorithm, and adjustment for relevant confounders. The TF2 framework provides a clinically relevant context for biomarker evaluation, as it represents real-world decision-making processes in pediatric critical care. Additionally, our findings demonstrate biological plausibility given the established role of RAAS dysregulation in critical illness and the mechanistic insights provided by previous work [21, 38]. However, limitations include the single-center design, which may limit generalizability to other pediatric intensive care populations with different case mixes and clinical practices. The opportunistic nature of enrollment, though it enhanced study feasibility, may have led to unintended selection bias. The moderate sample size may have limited our ability to detect smaller effect sizes or perform more complex multivariable analyses. We also lacked measurements of other RAAS components (angiotensin I, angiotensin II, aldosterone) that might provide additional mechanistic insights into the pathophysiology of RAAS dysregulation in critically ill children. Furthermore, the observational nature of our study precludes causal inferences about the relationship between renin elevation and kidney injury. The timing of renin measurement (within 48 h of admission) represents both a strength and a limitation of our study design. Early measurement allows for timely risk stratification and clinical decision-making when interventions may be most effective, which is clinically relevant for the TF2 algorithm's goal of early sAKI prediction. However, this single timepoint may not capture the dynamic nature of RAAS activation during critical illness, and serial measurements might provide additional prognostic information.

In conclusion, serum direct renin concentration shows promise as a complementary biomarker for refining sAKI risk stratification in critically ill children. Its greatest clinical performance value lies in enhancing the specificity and PPV of existing prediction models, particularly for identifying patients at the highest risk for sAKI. The progressive increase in renin levels across TF2 algorithm branches supports the biological rationale for RAAS dysregulation in AKI pathophysiology in critically ill children. Additionally, the association between elevated renin and mortality suggests broader prognostic utility beyond kidney-specific outcomes. Future research priorities should include external validation in multicenter cohorts, optimization of renin measurement timing and thresholds, exploration of serial renin measurements to capture dynamic RAAS changes, and investigation of targeted therapies based on RAAS profiling in pediatric populations. These findings represent an important step toward precision medicine approaches for sAKI prediction and management in PICU settings.

## Supplementary Information

Below is the link to the electronic supplementary material.

Graphical abstract (PPTX 248 KB)

ESM1 (PDF 307 KB)

## Data Availability

Deidentified datasets analyzed during the current study are available from the corresponding author on reasonable request.
