# Supplementary material for: Serum renin levels refine acute kidney injury prediction in critically ill children
Source: Pediatr Nephrol. 2025 Nov 22;41(4):1203–11. doi: 10.1007/s00467-025-07061-0 (PMC12953286; doi:10.1007/s00467-025-07061-0)
Supplement: Supplementary file 1 — Graphical abstract (PPTX 248 KB) [file 467_2025_7061_MOESM1_ESM.pptx]

## Slide 1
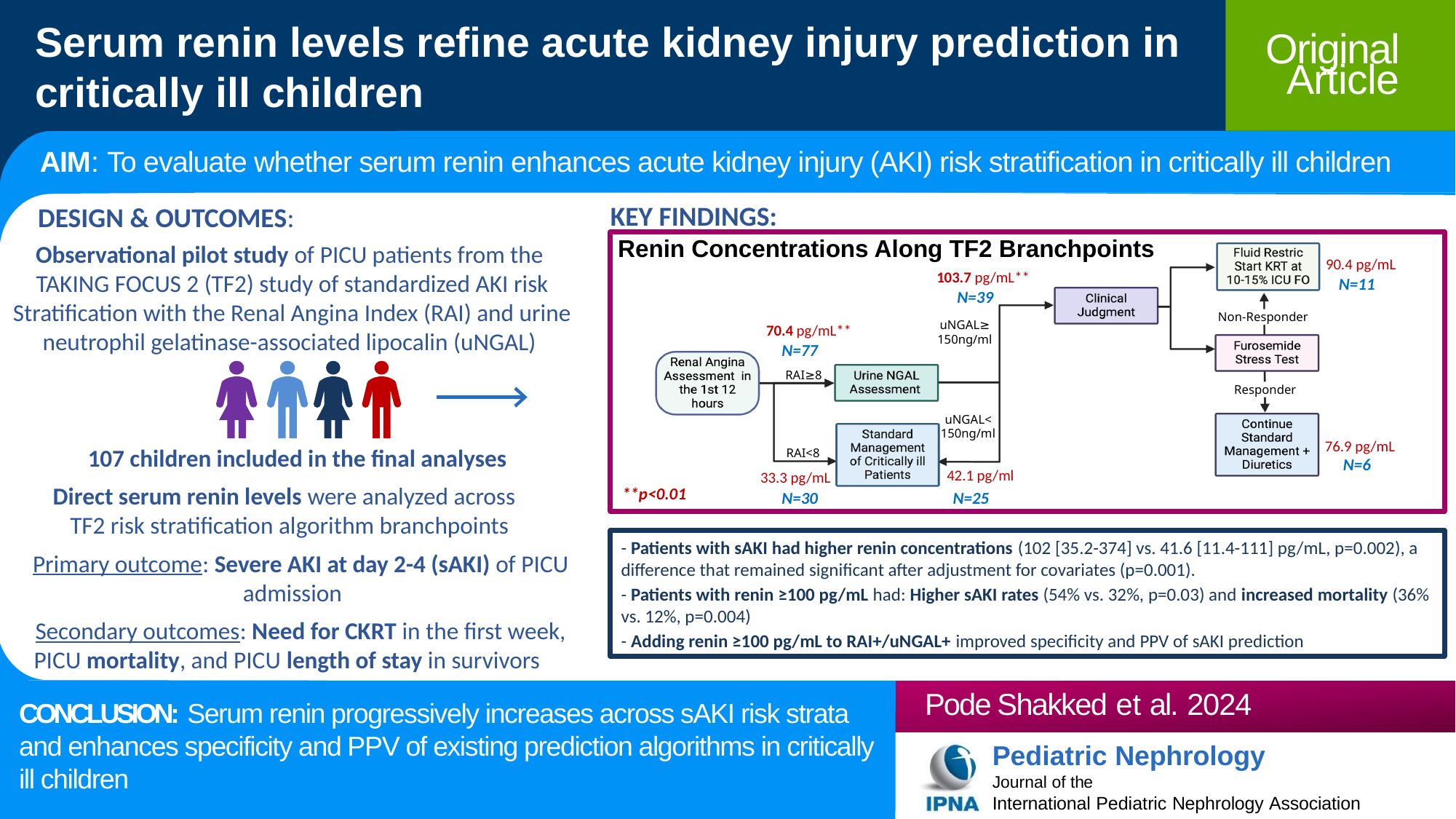

# Serum renin levels refine acute kidney injury prediction in critically ill children
Original Article
AIM: To evaluate whether serum renin enhances acute kidney injury (AKI) risk stratification in critically ill children
KEY FINDINGS:
DESIGN & OUTCOMES:
Renin Concentrations Along TF2 Branchpoints
Non-Responder
uNGAL≥
150ng/ml
RAI≥8
Responder
uNGAL<
150ng/ml
RAI<8
103.7 pg/mL**
90.4 pg/mL
70.4 pg/mL**
76.9 pg/mL
42.1 pg/ml
33.3 pg/mL
N=11
N=39
N=77
N=6
N=25
N=30
**p<0.01
Observational pilot study of PICU patients from the
TAKING FOCUS 2 (TF2) study of standardized AKI risk
Stratification with the Renal Angina Index (RAI) and urine neutrophil gelatinase-associated lipocalin (uNGAL)
 107 children included in the final analyses
Direct serum renin levels were analyzed across
TF2 risk stratification algorithm branchpoints
 Primary outcome: Severe AKI at day 2-4 (sAKI) of PICU admission
 Secondary outcomes: Need for CKRT in the first week, PICU mortality, and PICU length of stay in survivors
- Patients with sAKI had higher renin concentrations (102 [35.2-374] vs. 41.6 [11.4-111] pg/mL, p=0.002), a difference that remained significant after adjustment for covariates (p=0.001).
- Patients with renin ≥100 pg/mL had: Higher sAKI rates (54% vs. 32%, p=0.03) and increased mortality (36% vs. 12%, p=0.004)
- Adding renin ≥100 pg/mL to RAI+/uNGAL+ improved specificity and PPV of sAKI prediction
Pode Shakked et al. 2024
CONCLUSION: Serum renin progressively increases across sAKI risk strata and enhances specificity and PPV of existing prediction algorithms in critically ill children
Pediatric Nephrology
Journal of the
International Pediatric Nephrology Association
