# Supplementary material for: Serum renin levels refine acute kidney injury prediction in critically ill children
Source: Pediatr Nephrol. 2025 Nov 22;41(4):1203–11. doi: 10.1007/s00467-025-07061-0 (PMC12953286; doi:10.1007/s00467-025-07061-0)
Supplement: Supplementary file 2 — (PDF 307 KB) [file 467_2025_7061_MOESM2_ESM.pdf]

Online Supplement

Serum Renin Levels Refine Acute Kidney Injury Prediction in Critically Ill Children

Naomi Pode Shakked, MD, PhD[1,2,3], Giovanni Ceschia, MD[3], James E. Rose[1], Kelli A Krallman[1], Stuart L. Goldstein, MD[1,6], Natalja L. Stanski, MD, MS[1,6].

[1]Cincinnati Children's Hospital Medical Center, 3333 Burnet Ave, Cincinnati, OH, USA 45208

[2]Pediatric Nephrology Unit, Dana Dwek Children's Hospital, Tel Aviv Medical Center, Tel Aviv, Israel

[4]Gray Faculty of Medical & Health Sciences, Tel-Aviv University, Tel Aviv, Israel

[5]Pediatric Nephrology Unit, Department of Women's and Children's Health, University-Hospital of Padova, Via Giustiniani 3, 35128 Padova, Italy

[6]Department of Pediatrics, University of Cincinnati College of Medicine, 3230 Eden Ave, Cincinnati, OH, USA 45267

Corresponding Author:

Natalja L. Stanski, MD

Email: natalja.stanski@cchmc.org

Figure S1: The TAKING FOCUS 2 Algorithm from the original study.

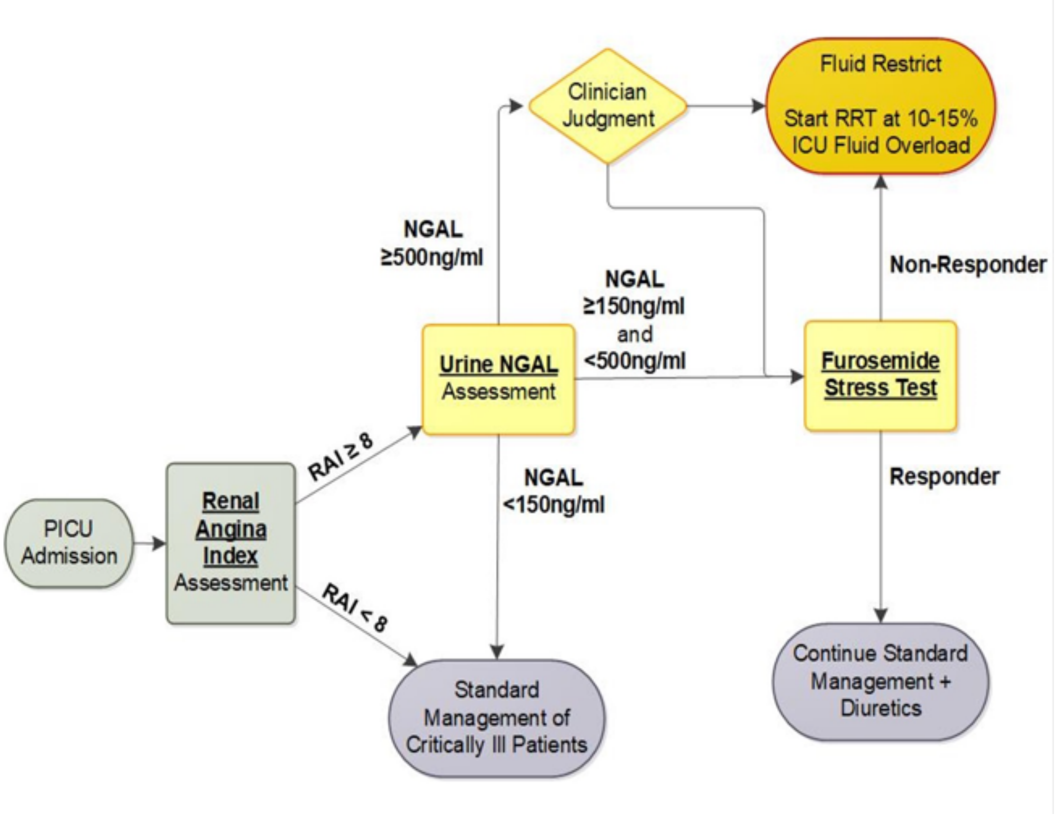

Figure S2: The Renal Angina Index. Risk and injury scores are multiplied to give the RAI score, with a score greater or equal to 8 being high risk for Day 2-4 severe acute kidney injury.

Risk Strata: Select highest that applies

Risk Criteria	Score
PICU Admission	1
History of transplantation (solid organ or stem cell)	3
Mechanical ventilation AND/OR vasoactive support	5

Injury Strata: Select highest that applies

SCr>Baseline	or % FO	Score
Decreased or no change	<5%	1
>1x – 1.49x	5-10%	2
1.5x – 1.99x	10-15%	4
≥ 2x	>15%	8

Risk x Injury = RAI
(Scores: 1-40)
